# Racial and Ethnic Disparities in Incidence of SARS-CoV-2 Infection, 22 US States and DC, January 1–October 1, 2020 

**DOI:** 10.3201/eid2705.204523

**Published:** 2021-05

**Authors:** NaTasha D. Hollis, Wen Li, Miriam E. Van Dyke, Gibril J. Njie, Heather M. Scobie, Erin M. Parker, Ana Penman-Aguilar, Kristie E.N. Clarke

**Affiliations:** Centers for Disease Control and Prevention, Atlanta, Georgia, USA (N.D. Hollis, W. Li, M.E. Van Dyke, G.J. Njie, H.M. Scobie, E. Parker, A. Penman-Aguilar, K.E.N. Clarke);; Commissioned Corps of the US Public Health Service, Atlanta (N.D. Hollis, H.M. Scobie, E.M. Parker, K.E.N. Clarke)

**Keywords:** coronavirus, infection, ethnicity, race, SARS-CoV-2, COVID-19, respiratory infections, severe acute respiratory syndrome coronavirus 2, 2019 novel coronavirus disease, coronavirus disease, zoonoses, viruses, coronaviruses, United States, disparities, inequities

## Abstract

We examined disparities in cumulative incidence of severe acute respiratory syndrome coronavirus 2 by race/ethnicity, age, and sex in the United States during January 1–October 1, 2020. Hispanic/Latino and non-Hispanic Black, American Indian/Alaskan Native, and Native Hawaiian/other Pacific Islander persons had a substantially higher incidence of infection than non-Hispanic White persons.

Health disparities among racial/ethnic minority groups in the United States are closely related to structural inequities in social determinants of health. Some racial/ethnic minority groups have disproportionate rates of underlying conditions that increase the risk for severe illness from coronavirus disease (COVID-19) (*1*,*2*). Certain groups are overrepresented in occupations that require public contact, have crowded conditions, or are unamenable to telework, increasing the risk for exposure to severe acute respiratory infection coronavirus 2 (SARS-CoV-2), the virus that causes COVID-19 (*3*,*4*). Structural inequities in housing, education, wealth, and healthcare access also increase disparities in infection and COVID-related illness and death (*5*–*8*).

We conducted an intersectional analysis by race/ethnicity, age, and sex to identify disparities in SARS-CoV-2 incidence using data from multiple US jurisdictions. Monitoring these disparities is critical for guiding action to reduce health inequities.

## The Study

We analyzed SARS-CoV-2 infections reported to the Centers for Disease Control and Prevention (https://data.cdc.gov/browse?tags=covid-19) by jurisdictional health departments. To minimize information bias, we included only jurisdictions reporting >30% of cases (https://protect-public.hhs.gov) and >70% completeness of race/ethnicity data of cases during January 1–October 1, 2020. We analyzed data on race/ethnicity, age, and sex in 1,751,627 cases from 22 US states and the District of Columbia (Table).

**Table:** Incidence of severe acute respiratory syndrome coronavirus 2 infections by sex, race/ethnicity, and age group, 22 US states and District of Columbia, January 1–October 1, 2020*

Characteristic	No. (%), n = 1,751,627†	Cumulative incidence (95% CI)‡§	Cumulative incidence ratio (95% CI)§
Sex			
F	898,970 (51.7)	1,734 (1,730–1,737)	Referent
M	841,487 (48.3)	1,672 (1,668–1,675)	0.96 (0.96–0.97)
Race and ethnicity¶			
Non-Hispanic White	657,437 (47.7)	935 (933–938)	Referent
Non-Hispanic Black	225,477 (16.4)	1,974 (1,965–1,982)	2.11 (2.10–2.12)
Non-Hispanic Asian	33,703 (2.4)	874 (865–884)	0.93 (0.92–0.95)
Non-Hispanic multiple races	22,650 (1.6)	957 (944–969)	1.02 (1.01–1.04)
Non-Hispanic American Indian or Alaska Native	19,259 (1.4)	2,274 (2,242–2,306)	2.43 (2.40–2.47)
Non-Hispanic Native Hawaiian or other Pacific Islander	7,226 (0.5)	2,693 (2,631–2,755)	2.88 (2.81–2.95)
Hispanic/Latino	375,418 (27.3)	2,860 (2,850–2,869)	3.06 (3.05–3.07)
Non-Hispanic other	36,104 (2.6)	NA	NA
Age group, y			
<19	191,303 (11.5)	774 (770–777)	0.33 (0.33–0.34)
20–34	473,627 (28.4)	2,316 (2,310–2,323)	Referent
35–44	270,405 (16.2)	2,146 (2,138–2,154)	0.93 (0.92–0.93)
45–54	258,400 (15.5)	2,060 (2,052–2,068)	0.89 (0.89–0.89)
55–64	216,848 (13.0)	1,591 (1,584–1,597)	0.69 (0.68–0.69)
65–74	128,348 (7.7)	1,220 (1,213–1,226)	0.53 (0.52–0.53)
75–84	74,539 (4.5)	1,366 (1,356–1,376)	0.59 (0.59–0.59)
>85	51,472 (3.1)	2,283 (2,263–2,303)	0.99 (0.98–0.99)

*Data from District of Columbia and 22 US states: Alaska, Arkansas, Florida, Hawaii, Iowa, Kansas, Massachusetts, Maine, Michigan, Minnesota, Mississippi, Montana, Nebraska, New Hampshire, New Mexico, Nevada, Ohio, Oregon, Tennessee, Utah, Vermont, and Wisconsin. Data from Data Collation and Integration for Public Health Event Responses platform (https://data.cdc.gov/browse?tags=covid-19). NA, not available. †Missing sex data for 11,170 persons; race/ethnicity data for 374,353 persons; and age data for 86,685 persons (not included in percentage calculations). ‡Cases per 100,000 persons. Population denominators from 2019 US Census (Annual County Resident Population Estimates by Age, Sex, Race, and Hispanic Origin, https://www.census.gov/programs-surveys/popest/technical-documentation/file-layouts.html). §Calculated using a normal approximation (Xu J, Kockanek KD, Murphy SL, Tejada-Vera B. Deaths: final data for 2007. National Center for Health Statistics. 2010 [cited 2020 Oct 16]. https://www.cdc.gov/nchs/data/nvsr/nvsr58/nvsr58_19.pdf). ¶No measures were calculated for 36,104 Non-Hispanic persons of other races because of lack of population denominator information from US Census Bureau.

We determined cumulative incidence of infection per 100,000 population and cumulative incidence ratios (CIRs) with 95% CIs by race/ethnicity, age, and sex. Patients were grouped as Hispanic or Latino (Hispanic), non-Hispanic American Indian or Alaska Native (AIAN), non-Hispanic Black or African American (Black), non-Hispanic Asian (Asian), non-Hispanic Native Hawaiian or other Pacific Islander (NHOPI), non-Hispanic White (White), or non-Hispanic of multiple races (multiple race). Of Hispanic persons in this sample, 53.8% identified as White, 33.2% as persons of multiple or other races, 1.7% as Black, 0.2% as Asian, and 0.2% as NHOPI; 10.5% of Hispanic persons were of unknown race. We used population denominators from the 2019 US Census (Annual County Resident Population Estimates by Age, Sex, Race, and Hispanic Origin, https://www.census.gov/programs-surveys/popest/technical-documentation/file-layouts.html). We considered CIR 95% CIs excluding 1.0 to be significant. We assessed differences in rates by sex after adjusting for race/ethnicity and age using Analysis of Variance. We conducted statistical analyses using R version 4.0.0 (*9*). This study was conducted in accordance with applicable federal law and Centers for Disease Control and Prevention policy [45 Code of Federal Regulations part 46.102(l)(2)].

We found that most racial/ethnic minority groups had significantly higher cumulative incidence of SARS-CoV-2 than did White persons (Table). Cumulative incidence ranged from 874 (95% CI 865–884)/100,000 population in Asian persons to 2,860 (95% CI 2,850–2,869)/100,000 population in Hispanic persons. CIRs were significantly higher among Black (2.11), AIAN (2.43), NHOPI (2.88), and Hispanic persons (3.06) compared with White persons; the CIR was nominally but significantly different for multiple race (1.02) and Asian persons (0.93). Cumulative incidence for men compared with women, when adjusted for both race/ethnicity and age, was similar (p = 0.982; data not shown).

**Figure 1:**
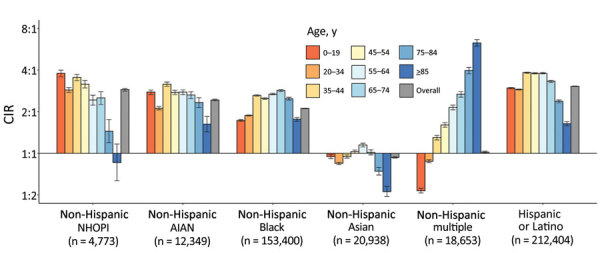
CIRs of severe acute respiratory syndrome coronavirus 2 among persons of different racial/ethnic groups compared with non-Hispanic White persons, 22 US states and the District of Columbia, January 1–October 1, 2020. Ratios are displayed on binary logarithmic scale; error bars indicate 95% CIs (Appendix Table 1). CIRs are displayed on binary logarithmic scale; error bars indicate 95% CIs. CIRs with error bars not crossing the origin (1:1) are significant (p<0.05). AIAN, American Indian or Alaska Native; CIRs, cumulative incidence ratios; NHOPI, Native Hawaiian or other Pacific Islander.

Cumulative incidence of SARS-CoV-2 was significantly higher among most racial/ethnic minority groups than among White persons of the same age group (Figure 1; Appendix Table 1). Among Asian persons <45 or >75 years of age, CIRs were lower (0.53–0.95) than among White persons. Among multiple race persons, results varied by age: CIRs were significantly lower among those <19 years of age (CIR 0.54, 95% CI 0.52–0.56) and 20–34 years of age (CIR 0.88, 95% CI 0.86–0.90) but ≈4–6 times higher among those >75 years of age. Black, AIAN, NHOPI (except for persons aged >85), and Hispanic persons had CIRs of 1.45–3.83 by age group.

**Figure 2:**
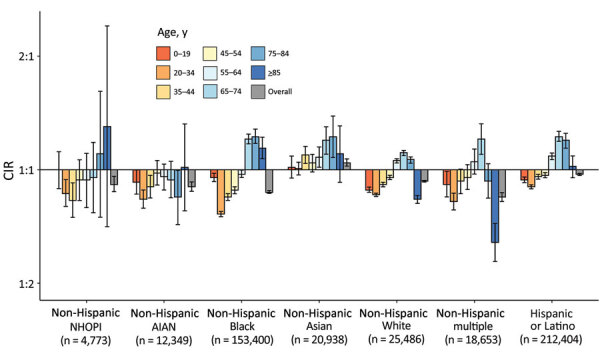
CIRs of severe acute respiratory syndrome coronavirus 2 for male sex, compared with female sex, 22 US states and District of Columbia, January 1–October 1, 2020. Ratios are displayed on binary logarithmic scale; error bars indicate 95% CIs (Appendix Table 2). CIRs are displayed on binary logarithmic scale; error bars indicate 95% CIs. CIRs with error bars not crossing the origin (1:1) are significant (p<0.05). AIAN, American Indian or Alaska Native; CIRs, cumulative incidence ratios; NHOPI, Native Hawaiian or other Pacific Islander.

We found differences in infection rates by sex within various racial/ethnic and age groups (CIRs 0.64–1.30) (Figure 2; Appendix Table 2). Overall, cumulative incidence among men in all racial/ethnic groups was significantly lower than among women (CIRs 0.85–0.97), with an exception among Asian men (CIR 1.05). Men who were Black and >65 years of age, multiple race and 65–74 years of age, and Hispanic or White and 55–84 years of age had a higher cumulative incidence than women. Among NHOPI and AIAN persons, cumulative incidence was significantly lower than for White persons only for men 20–44 years of age.

## Conclusions

Among >1.75 million persons with SARS-CoV-2 in 23 US jurisdictions during January 1–October 1, 2020, persons from most racial/ethnic minority groups had higher cumulative incidence than White persons. Hispanic persons had a 3.1-fold higher incidence and Black, AIAN, and NHOPI persons a >2-fold higher incidence of SARS-CoV-2 than did White persons. Racial/ethnic disparities varied by age group. Sex differences in cumulative incidence within racial/ethnic groups were less pronounced than disparities between racial/ethnic groups.

We found the highest incidence of infection among Hispanic persons, similar to findings of studies examining SARS-CoV-2 positivity rates in more limited US geographic areas (*6*,*10*–*12*). We also found high incidence among NHOPI persons. Previous analyses have rarely disaggregated NHOPI persons, preventing detection of disparities. Although previous studies have shown higher rates of severe COVID-19 illness among men, we observed lower infection rates among men overall (*1*,*13*).

Social determinants of health drive racial/ethnic disparities in disease incidence (*3*–*8*). For example, members of some racial/ethnic groups are overrepresented in the essential workforce and more likely to live in multigenerational or high-density housing, increasing the risk for SARS-CoV-2 exposure (https://www.cdc.gov/coronavirus/2019-ncov/community/health-equity/racial-ethnic-disparities/index.html). Outbreaks in some occupational settings have had racial/ethnic disparities in infection (*3*,*8*). Employers, community organizations, healthcare systems, public health agencies, and governments can act to reduce racial/ethnic disparities in COVID-19 incidence by implementing flexible, nonpunitive leave policies (e.g., paid sick leave); equitable access to testing and screening programs, personal protective equipment, and vaccines; and policies that encourage physical distancing (*14*). In addition, public health officials can tailor COVID-19 prevention messaging to the languages, and cultures of various racial/ethnic groups. Multisectoral partnerships could support COVID-19 mitigation strategies through initiatives that provide spaces for isolation or self-quarantine, safe transportation, free or reduced-cost broadband internet, and housing resources (*14*).

One limitation of this study is that underreporting to the Centers for Disease Control and Prevention database, which documented 78% of cases in selected jurisdictions, probably caused underestimates in calculated incidence. Second, selected jurisdictions comprise 31% of the US population; in these jurisdictions, NHOPI, White, AIAN, and multiple race persons are overrepresented and Asian, Hispanic, and Black persons underrepresented (Appendix Table 3). As a result, our findings are not nationally representative or generalizable. Third, we excluded persons of unknown race/ethnicity (24%) from incidence calculations. Among persons of unknown race/ethnicity, 33% specified race but not ethnicity; minority racial groups were overrepresented (Appendix Table 4). Fourth, cases among racial/ethnic minority groups might be underreported because of disparities in testing access (*15*). The third and fourth issues probably resulted in underestimation of racial/ethnic disparities. Finally, aggregation of NHOPI and Asian persons in >2 jurisdictions probably resulted in underestimating of incidence among NHOPI persons and overestimating among Asian persons.

In summary, documenting population-based racial/ethnic disparities in SARS-CoV-2 infection rates and how disparities vary by age and sex informs the development and implementation of equitable policies and intervention strategies. Strategies should prioritize collection and analysis of data relating to health equity and focus on mitigating disproportionate risks of exposure related to social determinants of health.

Appendix Additional data on racial and ethnic disparities in incidence of SARS-CoV-2 infections, United States, 2020.
