# Supplementary material for: Racial and Ethnic Disparities in Incidence of SARS-CoV-2 Infection, 22 US States and DC, January 1–October 1, 2020
Source: Emerg Infect Dis. 2021 May;27(5):1477–81. doi: 10.3201/eid2705.204523 (PMC8084494; doi:10.3201/eid2705.204523)
Supplement: Appendix — Additional data on racial and ethnic disparities in incidence of SARS-CoV-2 infections, United States, 2020. [file 20-4523-Techapp-s1.pdf]

Article DOI: https://doi.org/10.3201/eid2705.204523

Racial and Ethnic Disparities in Incidence of SARS-CoV-2 Infection, 22 US States and DC, January 1–October 1, 2020

Appendix

Appendix Table 1. Cumulative incidence of severe acute respiratory syndrome coronavirus 2 infection and cumulative incidence ratio (CIR) by race/ethnicity and age group, 22 US states and District of Columbia, January 1–October 1, 2020

Race/ethnicity†	Cumulative incidence (95% CI)‡§	CIR (95% CI)§	Cumulative incidence (95% CI)‡§	CIR (95% CI)§
Age, y	≤19		65–74	
Non-Hispanic NHOPI	1,587 (1,498–1,676)	3.78 (3.57–4.00)	1,843 (1,645–2,040)	2.51 (2.25–2.79)
Non-Hispanic AIAN	1,161 (1,118–1,203)	2.76 (2.66–2.87)	1,936 (1,829–2,043)	2.63 (2.49–2.78)
Non-Hispanic Black	727 (718–736)	1.73 (1.70–1.76)	2,088 (2,057–2,119)	2.84 (2.79–2.89)
Non-Hispanic White	420 (417–423)	Ref	735 (730–741)	Ref
Non-Hispanic Asian	398 (385–411)	0.95 (0.92–0.98)	748 (717–779)	1.02 (0.97–1.06)
Non-Hispanic Multiple	226 (217–234)	0.54 (0.52–0.56)	1,962 (1,871–2,053)	2.67 (2.55–2.80)
Hispanic/Latino	1,243 (1,233–1,254)	2.96 (2.93–2.99)	2,432 (2,395–2,469)	3.31 (3.25–3.36)
Age, y	20–34		75–84	
Non-Hispanic NHOPI	3,522 (3,378–3,666)	2.87 (2.75–2.99)	1,327 (1,072–1,582)	1.45 (1.19–1.75)
Non-Hispanic AIAN	2,601 (2,529–2,673)	2.12 (2.06–2.18)	2,136 (1,963–2,310)	2.33 (2.14–2.52)
Non-Hispanic Black	2,302 (2,284–2,320)	1.88 (1.86–1.89)	2,281 (2,231–2,330)	2.48 (2.43–2.54)
Non-Hispanic White	1,228 (1,222–1,234)	Ref	918 (909–927)	Ref
Non-Hispanic Asian	1,037 (1,017–1,057)	0.84 (0.83–0.86)	681 (638–724)	0.74 (0.70–0.79)
Non-Hispanic Multiple	1,078 (1,050–1,105)	0.88 (0.86–0.90)	3,638 (3,450–3,827)	3.96 (3.76–4.18)
Hispanic/Latino	3,551 (3,530–3,573)	2.89 (2.87–2.92)	2,179 (2,130–2,228)	2.37 (2.32–2.43)
Age, y	35–44		≥85	
Non-Hispanic NHOPI	3,473 (3,290–3,656)	3.53 (3.35–3.72)	1,430 (992–1,867)	0.86 (0.63–1.17)
Non-Hispanic AIAN	3,112 (3,005–3,219)	3.16 (3.06–3.28)	2,705 (2,358–3,051)	1.63 (1.43–1.85)
Non-Hispanic Black	2,574 (2,548–2,600)	2.62 (2.59–2.65)	2,929 (2,838–3,019)	1.76 (1.71–1.82)
Non-Hispanic White	983 (977–990)	Ref	1,661 (1,643–1,679)	Ref
Non-Hispanic Asian	933 (909–958)	0.95 (0.92–0.97)	879 (805–952)	0.53 (0.49–0.58)
Non-Hispanic Multiple	1,277 (1,230–1,325)	1.30 (1.25–1.35)	10,443 (9,904–10,982)	6.29 (5.96–6.63)
Hispanic/Latino	3,763 (3,735–3,790)	3.83 (3.79–3.86)	2,722 (2,634–2,809)	1.64 (1.58–1.69)
Age, y	45–54		Overall	
Non-Hispanic NHOPI	3,213 (3,013–3,413)	3.16 (2.97–3.37)	2,693 (2,631–2,755)	2.88 (2.81–2.95)
Non-Hispanic AIAN	2,798 (2,694–2,902)	2.75 (2.65–2.86)	2,274 (2,242–2,306)	2.43 (2.40–2.47)
Non-Hispanic Black	2,528 (2,501–2,555)	2.49 (2.46–2.52)	1,974 (1,965–1,982)	2.11 (2.10–2.12)
Non-Hispanic White	1,016 (1,009–1,023)	Ref	935 (933–938)	Ref
Non-Hispanic Asian	1,045 (1,017–1,073)	1.03 (1.00–1.06)	874 (865–884)	0.93 (0.92–0.95)
Non-Hispanic Multiple	1,630 (1,569–1,692)	1.60 (1.54–1.67)	957 (944–969)	1.02 (1.01–1.04)
Hispanic/Latino	3,846 (3,815–3,876)	3.79 (3.75–3.82)	2,860 (2,850–2,869)	3.06 (3.05–3.07)
Age, y	55–64			
Non-Hispanic NHOPI	2,083 (1,911–2,256)	2.42 (2.23–2.63)		
Non-Hispanic AIAN	2,380 (2,286–2,474)	2.77 (2.66–2.88)		
Non-Hispanic Black	2,309 (2,283–2,335)	2.68 (2.65–2.72)		
Non-Hispanic White	861 (855–866)	Ref		
Non-Hispanic Asian	987 (957–1,018)	1.15 (1.11–1.18)		
Non-Hispanic Multiple	1,846 (1,776–1,917)	2.14 (2.06–2.23)		
Hispanic/Latino	3,267 (3,234–3,300)	3.80 (3.75–3.84)		

*Data from District of Columbia and 22 US states: Alaska, Arkansas, Florida, Hawaii, Iowa, Kansas, Massachusetts, Maine, Michigan, Minnesota, Mississippi, Montana, Nebraska, New Hampshire, New Mexico, Nevada, Ohio, Oregon, Tennessee, Utah, Vermont, and Wisconsin. Data from Data Collation and Integration for Public Health Event Responses platform (https://data.cdc.gov/browse?tags=covid-19). AIAN, American Indian or Alaska Native; CIR, cumulative incidence ratio; NHOPI, Native Hawaiian or other Pacific Islander.
†The study participants comprised 7,266 non-Hispanic NHOPI; 19,259 non-Hispanic AIAN; 225,477 non-Hispanic Black; 657,437 non-Hispanic White; 33,703 non-Hispanic Asian; 22,650 non-Hispanic Multiple; and 375,418 Hispanic/Latino persons.
‡Cases per 100,000 persons. Population denominators from 2019 US Census (Annual County Resident Population Estimates by Age, Sex, Race, and Hispanic Origin, https://www.census.gov/programs-surveys/popest/technical-documentation/file-layouts.html).
§Calculated using a normal approximation.

Appendix Table 2. Cumulative incidence ratio (CIR) of severe acute respiratory syndrome coronavirus 2 among men compared with women by age group and race/ethnicity, 22 US states and District of Columbia, January 1–October 1, 2020

Race/ethnicity	CIR (95% CI)†	CIR (95% CI)†
Age, y	≤19	65–74
Non-Hispanic Asian	1.02 (0.95–1.09)	1.20 (1.10–1.30)
Non-Hispanic NHOPI	1.00 (0.89–1.12)	0.95 (0.77–1.18)
Non-Hispanic Black	0.95 (0.93–0.98)	1.21 (1.17–1.24)
Non-Hispanic AIAN	0.93 (0.86–1.00)	0.94 (0.84–1.05)
Non-Hispanic Multiple	0.92 (0.85–0.99)	1.21 (1.10–1.32)
Non-Hispanic White	0.88 (0.87–0.90)	1.11 (1.09–1.12)
Hispanic/Latino	0.94 (0.92–0.96)	1.23 (1.19–1.26)
Age, y	20–34	75–84
Non-Hispanic Asian	1.01 (0.97–1.05)	1.22 (1.08–1.39)
Non-Hispanic NHOPI	0.87 (0.80–0.94)	1.10 (0.75–1.61)
Non-Hispanic Black	0.76 (0.75–0.78)	1.23 (1.17–1.28)
Non-Hispanic AIAN	0.84 (0.79–0.88)	0.84 (0.72–1.00)
Non-Hispanic Multiple	0.82 (0.78–0.87)	0.93 (0.84–1.04)
Non-Hispanic White	0.86 (0.85–0.87)	1.06 (1.04–1.08)
Hispanic/Latino	0.90 (0.89–0.91)	1.19 (1.14–1.25)
Age, y	35–44	≥85
Non-Hispanic Asian	1.09 (1.04–1.15)	1.10 (0.93–1.31)
Non-Hispanic NHOPI	0.83 (0.75–0.92)	1.30 (0.71–2.40)
Non-Hispanic Black	0.85 (0.83–0.86)	1.14 (1.07–1.22)
Non-Hispanic AIAN	0.90 (0.84–0.97)	1.01 (0.78–1.32)
Non-Hispanic Multiple	0.93 (0.86–1.00)	0.64 (0.57–0.72)
Non-Hispanic White	0.91 (0.90–0.93)	0.83 (0.81–0.85)
Hispanic/Latino	0.96 (0.94–0.97)	1.02 (0.95–1.09)
Age, y	45–54	Overall
Non-Hispanic Asian	1.04 (0.99–1.10)	1.05 (1.02–1.07)
Non-Hispanic NHOPI	0.94 (0.83–1.06)	0.92 (0.87–0.96)
Non-Hispanic Black	0.88 (0.86–0.90)	0.87 (0.87–0.88)
Non-Hispanic AIAN	0.98 (0.91–1.06)	0.90 (0.88–0.93)
Non-Hispanic Multiple	0.96 (0.89–1.03)	0.85 (0.82–0.87)
Non-Hispanic White	0.95 (0.94–0.97)	0.93 (0.93–0.94)
Hispanic/Latino	0.97 (0.95–0.98)	0.97 (0.97–0.98)
Age, y	55–64	
Non-Hispanic Asian	1.08 (1.02–1.15)	
Non-Hispanic NHOPI	0.94 (0.79–1.10)	
Non-Hispanic Black	0.98 (0.95–1.00)	
Non-Hispanic AIAN	0.96 (0.88–1.04)	
Non-Hispanic Multiple	1.05 (0.97–1.13)	
Non-Hispanic White	1.06 (1.04–1.07)	
Hispanic/Latino	1.08 (1.06–1.11)	

*Data from District of Columbia and 22 US states: Alaska, Arkansas, Florida, Hawaii, Iowa, Kansas, Massachusetts, Maine, Michigan, Minnesota, Mississippi, Montana, Nebraska, New Hampshire, New Mexico, Nevada, Ohio, Oregon, Tennessee, Utah, Vermont, and Wisconsin. Data from Data Collation and Integration for Public Health Event Responses platform (https://data.cdc.gov/browse?tags=covid-19). Reference group: women. AIAN, American Indian or Alaska Native; CIR, cumulative incidence ratio; NHOPI, Native Hawaiian or other Pacific Islander.
†Calculated using a normal approximation.

Appendix Table 3. Racial/ethnic population distribution in study of disparities in severe acute respiratory syndrome coronavirus 2 infection, 22 US states and District of Columbia, January 1–October 1, 2020*

	Population, no. (%)	
Racial/ethnic group	All US states + District of Columbia	Study states + District of Columbia
Non-Hispanic White	197,309,822 (60.1)	70,293,994 (68.8)
Hispanic/Latino	60,572,237 (18.5)	13,128,453 (12.8)
Non-Hispanic Black	41,147,488 (12.5)	11,424,838 (11.2)
Non-Hispanic Asian	18,905,879 (5.8)	3,854,786 (3.8)
Non-Hispanic Multiple	7,273,281 (2.2)	2,367,411 (2.3)
Non-Hispanic American Indian or Alaska Native	2,434,908 (0.7)	846,922 (0.8)
Non-Hispanic Native Hawaiian or other Pacific Islander	595,908 (0.2)	268,314 (0.3)
Total	328,239,523 (100.0)	102,184,718 (100.0)

*Data from District of Columbia and 22 US states: Alaska, Arkansas, Florida, Hawaii, Iowa, Kansas, Massachusetts, Maine, Michigan, Minnesota, Mississippi, Montana, Nebraska, New Hampshire, New Mexico, Nevada, Ohio, Oregon, Tennessee, Utah, Vermont, and Wisconsin. Data from 2019 US Census (Annual County Resident Population Estimates by Age, Sex, Race, and Hispanic Origin, https://www.census.gov/programs-surveys/popest/technical-documentation/file-layouts.html). CIR, cumulative incidence ratio.

Appendix Table 4. Participants of unknown ethnicity in study on severe acute respiratory syndrome coronavirus 2 incidence, 22 US states and District of Columbia, January 1–October 1, 2020*

Race†	Persons of known race/ethnicity	Persons of unknown ethnicity but known race	Proportion of unknown ethnicity, %‡
Black	231,956	24,933	9.7
Multiple/other	183,359	35,493	16.2
Asian	34,499	4,486	11.5
American Indian or Alaska Native	20,711	2,760	11.8
Native Hawaiian or other Pacific Islander	7,813	773	9.0
Total non-White	478,338	68,445	12.5
White	859,368	68,733	7.4

*Table excludes 223,839 participants of unknown race and unknown ethnicity; 39,568 of unknown race and Hispanic/Latino ethnicity; and 13,336 of unknown race and non-Hispanic ethnicity.

†Race without regard to ethnicity. “Known” includes “Other race” (i.e., race was reported but could not be assigned to a category).

‡Persons of unknown ethnicity but known race out of the sum of “Persons of known race/ethnicity” and "Persons of unknown ethnicity but known race.”
